# A Cost-effectiveness Analysis of a Mobile Phone–Based Integrated HIV-Prevention Intervention Among Men Who Have Sex With Men in China: Economic Evaluation

**DOI:** 10.2196/38855

**Published:** 2022-11-02

**Authors:** Ke Yun, Jiaming Yu, Changyang Liu, Xinxin Zhang

**Affiliations:** 1 Department of Laboratory Medicine The First Affiliated Hospital of China Medical University Shenyang China; 2 Department of Hospital Infection Management The Fourth Affiliated Hospital of China Medical University Shenyang China; 3 Department of Ophthalmology Laboratory The Fourth Affiliated Hospital of China Medical University Shenyang China

**Keywords:** cost-effectiveness, digital intervention, men who have sex with men, mathematical model

## Abstract

**Background:**

Mobile phone–based digital interventions have been shown to be a promising strategy for HIV prevention among men who have sex with men (MSM).

**Objective:**

This study aimed to evaluate the cost-effectiveness of a mobile phone–based digital intervention for HIV prevention among MSM in China from the perspective of a public health provider.

**Methods:**

The cost-effectiveness of the mobile phone–based digital intervention was estimated for a hypothetical cohort of 10,000 HIV-negative MSM who were followed for 1 year. A model was developed with China-specific data to project the clinical impact and cost-effectiveness of two mobile phone–based digital strategies for HIV prevention among MSM. The intervention group received an integrated behavioral intervention that included 1) individualized HIV infection risk assessment, 2) recommendation of centers testing for HIV and other STIs, 3) free online order of condoms and HIV and syphilis self-test kits and 4) educational materials about HIV/AIDS. The control group was only given educational materials about HIV/AIDS. Outcomes of interest were the number of HIV infections among MSM averted by the intervention, intervention costs, cost per HIV infection averted by the mobile phone–based digital intervention, and quality-adjusted life-years (QALYs). Univariate and multivariate sensitivity analyses were also conducted to examine the robustness of the results.

**Results:**

It is estimated that the intervention can prevent 48 MSM from becoming infected with HIV and can save 480 QALYs. The cost of preventing 1 case of HIV infection was US $2599.87, and the cost-utility ratio was less than 0. Sensitivity analysis showed that the cost-effectiveness of the mobile phone–based digital intervention was mainly impacted by the average number of sexual behaviors with each sexual partner. Additionally, the higher the HIV prevalence among MSM, the greater the benefit of the intervention.

**Conclusions:**

Mobile phone–based digital interventions are a cost-effective HIV-prevention strategy for MSM and could be considered for promotion and application among high-risk MSM subgroups.

## Introduction

HIV infection confers a heavy public health burden worldwide [1,2]. In 2011, the Joint United Nations Programme on HIV/AIDS set a goal to increase financing to US $22 billion to $24 billion in order to prevent and control AIDS by 2015 [3]. At that time, the prevention and control of AIDS were put on a fast track to advance the 90-90-90 goal: 90% of all people living with HIV will know their HIV status, 90% of all people with diagnosed HIV infection will receive sustained antiretroviral therapy, and 90% of all people receiving antiretroviral therapy will have viral suppression. To achieve this goal, US $35 billion needed to be added to prevention-effort funding per year up to 2020 [3,4].

When policy makers for HIV prevention and control projects plan and assess AIDS prevention schemes, they need to balance the cost and effectiveness of various preventive efforts from behavioral studies that have transformed into community practices. There is an urgent unmet need for development of effective and cost-effective intervention strategies to prevent and control HIV in MSM. There have been many explorations on economic assessments of HIV behavioral interventions all over the world. For example, one study concluded that a video-based outpatient intervention effectively reduced the incidence of sexually transmitted infections (STIs) [5]. It was estimated that, on average, US $447,005 per year was spent offering 10,000 STI clinic patients the intervention, which equates to a cost per patient of US $43.30; the intervention prevented an average of 27.69 patients from acquiring HIV infection and saved US $5,544,408 in medical expenses [5]. Another study found that safer-sex lectures and skills training for MSM significantly increased condom use rates, and the incremental cost of the intervention was less than the cost-effectiveness threshold (ie, US $13,000), indicating cost savings [6]. In China, some studies have also demonstrated the cost-effectiveness of different HIV-prevention strategies (ie, advocacy interventions, peer education, outreach services, and intervention staff training), including a risk-related behavioral intervention among female sex workers [7], active HIV voluntary counseling and testing among the general population [8], and oral pre-exposure prophylaxis and expanded antiretroviral therapy among MSM [9].

Recently, the internet is becoming an essential platform for health behavior interventions and provides new opportunities to deliver information about HIV prevention for MSM [10]. In previous studies, we developed an HIV risk–prediction model for MSM [11] and comprehensive behavioral intervention strategies based on this model [12]. The comprehensive intervention strategies were shown to reduce the number of male sexual partners and increase the rate of condom use with casual partners among MSM through a randomized controlled trial [12]. However, participation in online projects is often not well controlled, resulting from the broad coverage of internet-based interventions, and can easily lead to cost overruns. Additionally, health economic evaluations regarding scaling up mobile phone–based digital interventions for risk reduction among MSM are lacking. Therefore, the aim of this study was to evaluate the health economic value of mobile phone–based digital intervention strategies among a larger number of MSM from the perspective of a public health provider.

## Methods

### Model Structure and Parameters

A widely used international model based on probability was used to project the number of HIV infections averted as well as the cost-effectiveness of two different intervention strategies among MSM [13]. The mathematical formula is defined as follows:

*P_HIV_* = 1 – {*P*[1 – *R*(1 – *FE*)]*^N^* + (1 – *P*)}*^M^*

where *P* represents the HIV prevalence among MSM, *R* is the risk of HIV infection during unprotected sex, and *E* is the protective effect of condoms. These three parameters were obtained from literature searches. In the formula, *F* is the rate of condom use during sexual behaviors, *N* represents each partner’s average number of sexual behaviors, and *M* represents the average number of sexual partners. These three parameters were obtained from the questionnaires completed during the clinical trial. The estimated value of each parameter was the annual average. The time interval studied and analyzed in this section was 1 year. A total of 10,000 MSM were included in the simulation.

### Epidemiological Data

The epidemiologic and behavioral parameters of HIV-infected patients are shown in Table 1 [12,14-20]. All the parameters above were estimated over an interval of 1 year. Critical model-fitting parameters, including HIV infection rate, HIV infectivity of one homosexual behavior, condom effectiveness, quality-adjusted life-years (QALYs) saved per HIV infection averted, lifetime medical costs after HIV infection, and the discount rate, were obtained by consulting the literature. According to clinical trial data, the average numbers of sexual partners in the intervention and control groups were 6.01 and 3.51, respectively. Condom use rates among MSM and their random sexual partners in the intervention and control groups were 0.86 and 0.70, respectively. According to the mathematical formula listed above, the probabilities of infection in the intervention and control groups were 0.002 and 0.007, respectively. The costs associated with the intervention and control groups were nearly equal. Moreover, one-third of the total costs were made up of labor and facility charges, which were the largest expenses for both groups.

**Table 1:** Model input parameters and costs.

Parameter			Value	Range	Data source
**Both groups**					
		HIV infection rate	0.073	—^a^	Qin et al [14]
		HIV infectivity rate of one homosexual behavior	0.008	0.002-0.02	Vittinghoff et al [15]
		Rate of condom effectiveness	0.95	0.69-0.99	Pinkerton and Abramson [16]
		The average number of sexual behaviors with each sexual partner, n	6	1.2-13.2	Zhang et al [17]
		QALYs^b^ saved per HIV infection averted, n	10	—	Hu et al [18]
		Lifetime medical costs after HIV infection (US $)	25,803.41	—	Wang et al [20]
		Discount rate, %	3	—	Wilde and Parke [19]
**Control group**					
	**Epidemiology**				
		Number of sexual partners, mean	6.01	—	Yun et al [12]
		Use rate of condoms for each sexual behavior	0.7	—	Yun et al [12]
		Infection probability	0.007	—	Calculated
	**Costs (US $)**				
		Recruitment spending	156.25	—	Observed
		Condoms and lubricant	—	—	Observed
		HIV self-test kits and supporting supplies	—	—	Observed
		HIV risk–assessment software development	—	—	Observed
		Human capital cost	843.75	—	Observed
		Communication fees and electricity charges	93.75	—	Observed
		Computers	1250	—	Observed
		Online survey and intervention system	—	—	Observed
		Office supplies (eg, paper and pens)	15.63	—	Observed
**Intervention group**					
	**Epidemiology**				
		Number of sexual partners, mean	3.51	—	Yun et al [12]
		Use rate of condoms for each sexual behavior	0.86	—	Yun et al [12]
		Infection probability	0.0022	—	Calculated
	**Costs (US $)**				
		Recruitment spending	156.25	—	Observed
		Condoms and lubricant	70.31	—	Observed
		HIV self-test kits and supporting supplies	93.75	—	Observed
		HIV risk–assessment software development	93.75	—	Observed
		Human capital cost	843.75	—	Observed
		Communication fees and electricity charges	93.75	—	Observed
		Computers	1250	—	Observed
		Online survey and intervention system	87.5	—	Observed
		Office supplies (eg, paper and pens)	15.63	—	Observed

^a^Not reported.

^b^QALY: quality-adjusted life-year.

### Intervention

**Figure 1:**
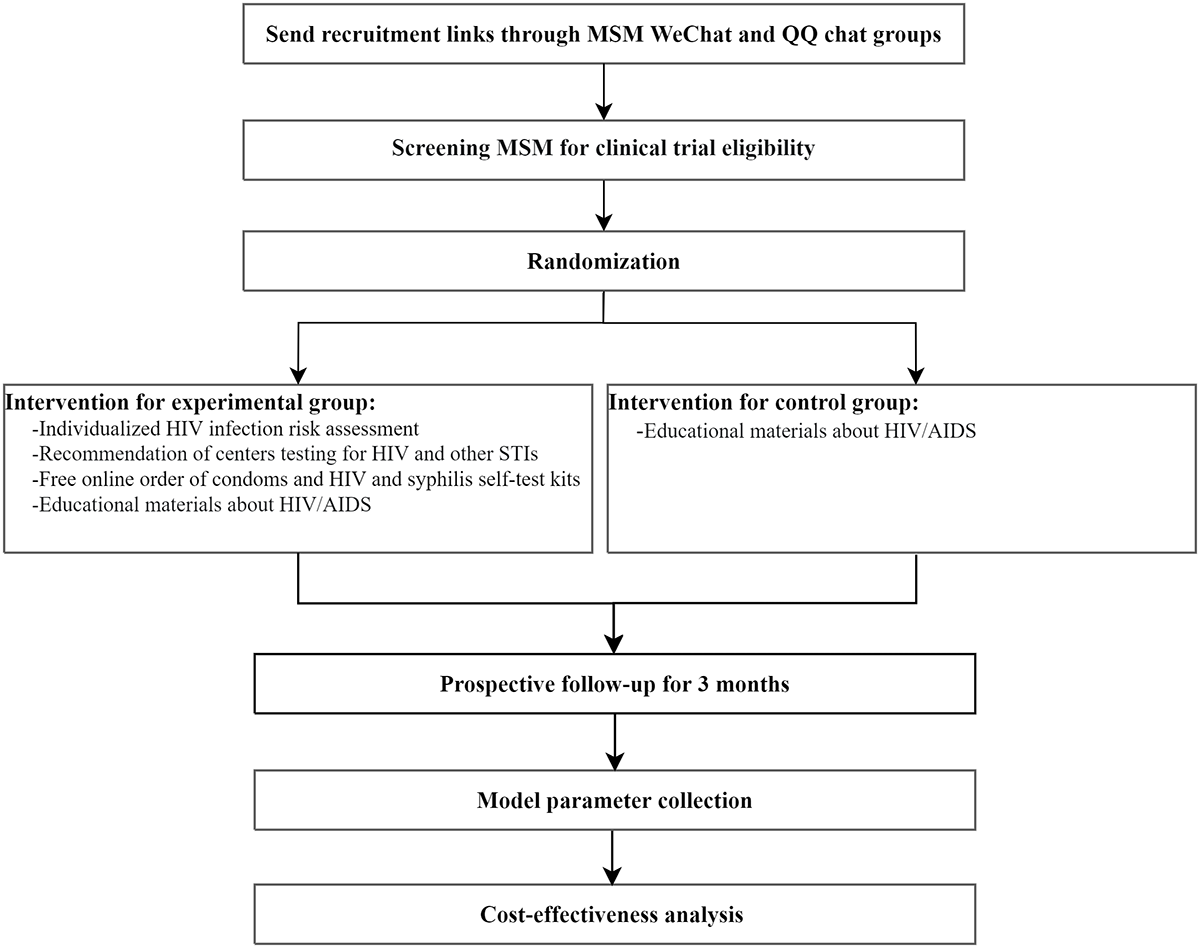
Cost-effectiveness analysis flowchart of the mobile phone–based digital intervention. MSM: men who have sex with men; STI: sexually transmitted infection.

These parameters were collected from a randomized, double-blind clinical trial, and the trial process is listed in Figure 1. With the assistance of a community-based organization, MSM were recruited from October 2017 to March 2018 through an advertisement in Tencent’s WeChat and QQ chat groups, which are popular virtual dating environments for MSM in China. The changes in HIV-related behavioral indicators in the intervention and control groups of MSM in the clinical trial were published previously [12]. Briefly, we prospectively evaluated the efficacy of two mobile phone–based digital strategies targeting MSM in China.

The intervention group received an integrated behavioral intervention promoted through WeChat, which included 1) individualized HIV infection risk assessment, 2) recommendation of centers testing for HIV and other STIs, 3) free online order of condoms and HIV and syphilis self-test kits and 4) educational materials about HIV/AIDS. The control group was only given educational materials about HIV/AIDS.

### Measurement of Costs

Costs were estimated from the perspective of a public health provider; only the direct costs used for the intervention were evaluated. The costs, which were estimated according to the clinical trial, included human capital costs, nongovernmental organization recruitment and referral fees, communication fees, electricity charges, condom and lubricant costs, HIV self-test kit costs, postage fees, computer costs, fees related to the online questionnaire system, compensation for completing questionnaires, HIV risk–assessment software deployment costs, and office supplies (eg, paper and pens).

### Estimation of QALYs Saved

The difference between the estimated values of behavioral parameters in the intervention and control groups was used to calculate the number of HIV-1 infections averted as a result of the intervention. QALYs represent the life-years obtained by the model multiplied by the definite utility value, shown as QALY = ∑WY, where W is the weight value and Y is the length of life. Quality of life–adjusted weights between 0 and 1 were used to calculate QALYs, also called health utility; this was the criterion used to assess health status and it reflected the severity of poor health. The total number of QALYs saved as a result of the intervention was calculated by the number of QALYs saved per HIV infection averted multiplied by the number of HIV infections averted. The cost-effectiveness ratio (CER) was one of the indicators evaluating economics that was used in this study. A lower ratio indicated that a lower cost was needed to gain one indicator effect unit. In the cost-utility ratio (CUR) analysis, an effect indicator combining life-years and health-related quality of life was performed. CUR, the ratio between the net cost of the intervention and the total QALYs saved, reflected the cost of a beneficial intervention. The incremental CER (ICER), whose units are US dollars per QALY, was also calculated to reflect the incremental cost associated with one additional unit of the effect measure.

### Sensitivity Analysis

As the values of many parameters were uncertain in the model, those of other parameters were controlled for consistency and to test the influence of parameter uncertainty on the final estimated results, which would help determine the essential factors affecting the cost effectiveness of the intervention. In this study, the one-way sensitivity analysis was mainly conducted to assess the average number of sexual behaviors with each sexual partner, HIV infectivity rate, QALYs saved per infection averted, HIV infection rate, rate of condom effectiveness, cost of offering intervention to 1 MSM (US $). Multiway sensitivity analysis was performed on the cost-effectiveness of the mobile phone–based digital intervention for HIV prevention as a function of intervention coverage and HIV prevalence among MSM.

### Judging Criteria

The ICER represents the incremental cost associated with 1 QALY saved. This measurement was used to determine which strategy was better. The criterion applied by the World Health Organization to determine whether interventions are cost-effective suggests that strategies evaluated with an ICER of less than 0 are both effective and cost-effective, interventions with an ICER of less than the average per capita gross domestic product (GDP) for a given country or region are considered very cost-effective, interventions with an ICER of less than 3-fold average per capita GDP are still considered cost-effective, and those that exceed this level are considered not cost-effective [21]. The average per capita GDP for China in 2017 was US $9293.49, which was used as the threshold to indicate whether the interventions were cost-effective in this study. A negative CUR suggests that the cost of a strategy in the intervention group is less than the cost of a strategy in the control group, which defines a cost-saving intervention strategy. Simply, the lower the CUR, the fewer health resources that are consumed.

**Ethics Approval**

The Ethics Review Committee of the First Affiliated Hospital of China Medical University in Shenyang, China, approved this study (approval No. 2018-175-2).

## Results

### Cost-effectiveness Analysis

The average annual cost to offer the intervention to 10,000 MSM was estimated to be US $124,837.50, with a cost per MSM of US $12.48. The model estimated that 70 of the 10,000 MSM would be infected with HIV without the intervention (ie, control group parameters). The model also estimated that 22 of the 10,000 MSM would be infected with HIV if they received the intervention (ie, intervention group parameters were applied). HIV infections could be averted in 48 persons per year with the intervention, with an average cost of US $2599.87 per infection averted. The intervention saved 480 QALYs per year, equaling a savings of US $980,529.61. Each QALY saved was accompanied by an incremental cost of about US $260.20, and the CUR was negative, indicating cost-effectiveness. Details are shown in Table 2.

**Table 2:** The 1-year cost-effectiveness analysis of a mobile phone–based digital intervention among 10,000 men who have sex with men.

Parameters			Value		Data source
**Intervention costs (US $)**					
	Recruiting advertisement fees	1562.50		Estimates from clinical trial data	
	Condoms and lubricant	28,125.00		Estimates from clinical trial data	
	HIV self-test kits and supporting supplies	37,500.00		Estimates from clinical trial data	
	Postage fees	37,500.00		Estimates from clinical trial data	
	Human capital costs	16,875.00		Estimates from clinical trial data	
	Communication fees and electricity charges	1875.00		Estimates from clinical trial data	
	Computers	1250.00		Estimates from clinical trial data	
	Online survey system	87.50		Estimates from clinical trial data	
	Office supplies (eg, paper and pens)	62.50		Estimates from clinical trial data	
	Annual cost	124,837.50		Estimates from clinical trial data	
	Cost of offering intervention to 1 MSM^a^	12.48		Estimates from clinical trial data	
**Intervention effect**					
	HIV infections averted, n	48		Calculated	
	QALYs^b^ saved by the intervention, n	480		Calculated	
	Medical costs saved by the intervention (US $)	980,529.61		Calculated	
	CER^c^	260.20		Calculated	
	Cost associated with 1 QALY saved (US $)	260.20		Calculated	
	CUR^d^	<0		Calculated	

^a^MSM: men who have sex with men.

^b^QALY: quality-adjusted life-year.

^c^CER: cost-effectiveness ratio.

^d^CUR: cost-utility ratio.

### Sensitivity Analysis

**Figure 2:**
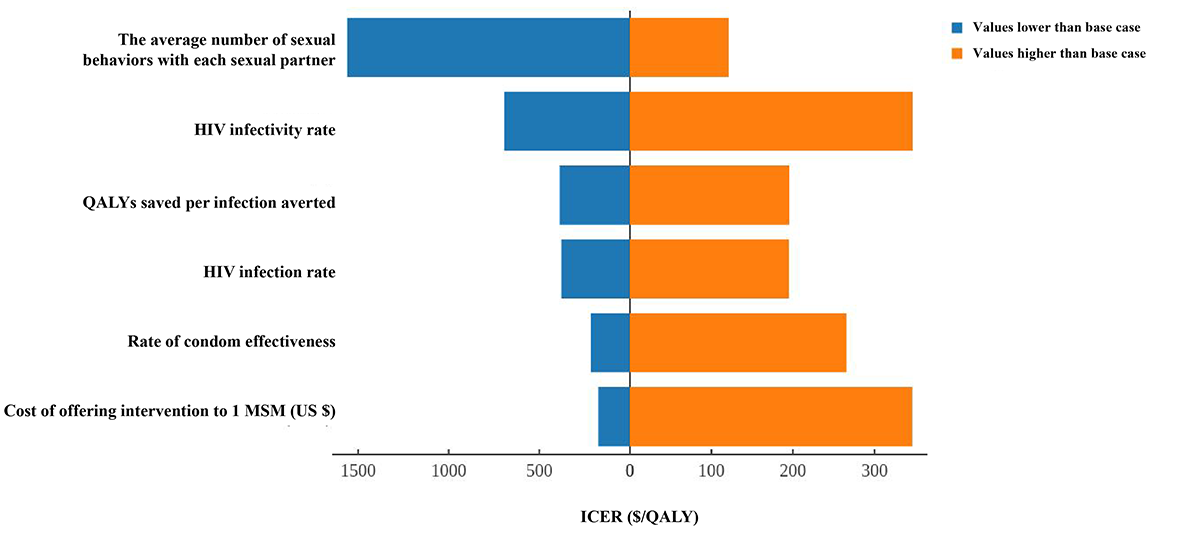
Tornado diagram of the one-way sensitivity analysis summarizing the effect of parameters on the ICER. The extent of the fluctuations in the parameters obtained from the literature is estimated by the 33% increase or decrease in the parameters reported in the literature. ICER: incremental cost-effectiveness ratio; MSM: men who have sex with men; QALY: quality-adjusted life-year.

Many parameters in the model were based on hypotheses and adjusted observed data, which led to various estimated outcomes under different situations; therefore, we performed a sensitivity analysis to test the effect of changes to the input parameters on the estimated outcomes of the model. The range of parameter fluctuations estimated according to the reported studies was adjusted to account for fluctuations of 33%. One-way sensitivity analyses showed that the number of infections averted was not sensitive to changes in most sexual behavior and epidemiologic parameters. Still, the average number of sexual behaviors with each sexual partner influenced the model’s outcomes to a certain extent. When the average number of sexual behaviors with each sexual partner was small, the cost associated with 1 QALY saved was US $1560.01. However, when the average number of sexual behaviors with each sexual partner was large, the cost associated with 1 QALY saved was US $121.17. These results indicated that the mobile phone–based digital intervention was more cost-effective among MSM who had more high-risk sexual behaviors (Figure 2).

**Figure 3:**
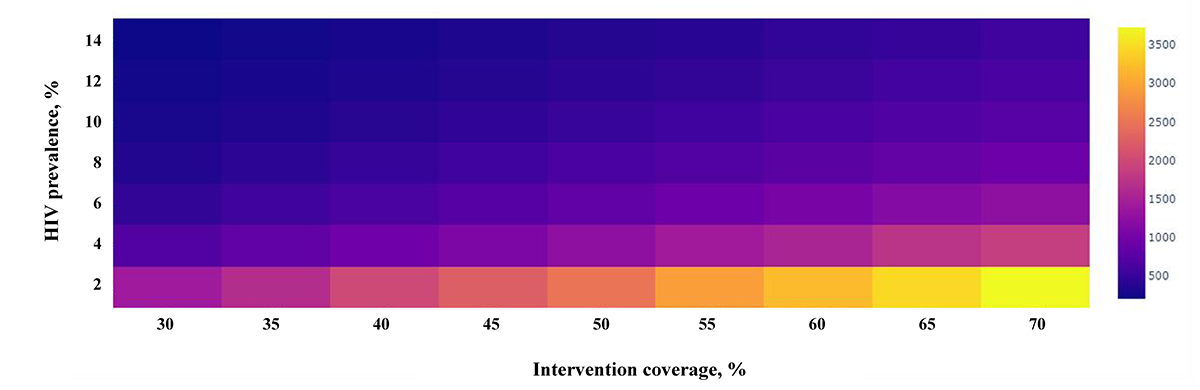
Multiway sensitivity analysis on the cost-effectiveness of the digital intervention for HIV prevention as a function of the intervention coverage and HIV prevalence among men who have sex with men. The colors signify the incremental cost-effectiveness ratios.

Multiway sensitivity analyses are shown in Figure 3, which showed ICER ranges for MSM as a function of intervention coverage and HIV prevalence. The cost-effectiveness of the mobile phone–based digital intervention depends on intervention coverage and HIV prevalence among MSM. A two-way sensitivity analysis showed that as MSM coverage increased, so did the cost, although the mobile phone–based digital intervention was still cost-effective in all simulated scenarios (HIV infection rate ranged from 0.02 to 0.14; intervention coverage ranged from 0.3 to 0.7). Additionally, the higher the HIV prevalence, the greater the benefit of the intervention.

## Discussion

### Principal Findings

HIV infection leads to substantial health, economic, and social consequences [22]. This study was the first to perform an economic evaluation of a mobile phone–based digital intervention in China. Overall, the intervention had high cost-effectiveness; the average number of sexual behaviors with each sexual partner and the intervention coverage affected the model’s outcomes. Since the number of people who avoided HIV infection as a result of the intervention cannot be directly measured, our study uses disease model prediction–based methods to estimate the number of people who avoided HIV infection as a result of the intervention. The cost-utility analysis used QALYs to consider both the length and quality of life, making it possible to compare different intervention strategies across the board. These findings provide a theoretical basis for decision-making regarding the government’s intervention policies.

This study found that although the effect of the online, personalized, risk-reduction intervention was slight, many cases of HIV infection can be avoided at the population level to achieve the goal of saving QALYs. Similar findings have been confirmed by other studies. For example, Zhang et al [23] have used the HIV phylogenetic tree to reveal the influence of individual HIV infection risk fluctuations on HIV transmission dynamics. They found that the change in the individual risk of HIV infection could change not only the proportion of HIV transmission among MSM but also the endemic equilibrium of HIV infection. These findings also suggest that interventions to reduce the risk of HIV infection at the individual level are important for controlling the HIV epidemic, and that appropriate targeted intervention strategies should be developed as soon as possible.

Compared with the study that evaluated the health economics of facility-based interventions among MSM in China [20], our intervention was more cost-effective. The difference may be explained by differences in intervention patterns. Compared with facility-based intervention strategies, internet-based interventions have the obvious advantages of high recruitment efficiency and low cost [10]. The essence of the intervention in this study was to convert offline consulting and testing into online risk self-assessment and self-testing (ie, facility-based testing or self-testing using testing strips). One of the reasons that this type of intervention strategy was cost-effective was that our study recommended individual intervention strategies and offered suggestions according to individual risk levels, leading to more precise resource allocation. In addition, internet-based interventions benefit hidden populations of MSM, especially young MSM [24,25], which is favorable for recruitment of large numbers of MSM. As a result, MSM benefit from improved access to health resources, expanded coverage of interventions, and improvements in the effectiveness of interventions [26]. In the future, we may consider building a real-time, interactive, big data intervention platform based on “Internet+,” which will continue to enrich the content and form of the intervention. At the same time, this will increase the publicity and education efforts of the intervention through the internet development platform in order to narrow the gap between the MSM population and HIV-prevention information. This will promote a more efficient flow of prevention resources in order to avoid redundancy and waste of health resources and will allow more MSM to take the initiative in participating in AIDS prevention and control.

A cost-utility analysis, believed to be the gold standard of economic evaluation, was conducted that took both length and quality of life into account regarding QALYs to make horizontal comparisons of different intervention strategies possible. Compared with other primary prevention strategies for HIV, the cost per HIV infection averted in our study (US $3337) was lower than that of a community-level HIV risk reduction intervention for women (US $65,000) [27], a video-based intervention model in STI clinics for men (US $21,486) [5], HIV-prevention skills training for MSM (US $4150.14) [6], and an internet-based video intervention (US $14,926). The reason why this intervention model is superior to the above strategies may be that with other intervention strategies, such as video education, nonvideo education, and condom distribution, there are significant differences in HIV risk behavior across these subgroups, and the emphasis on the use of health resources is also different. Additionally, the sensitivity analysis in our study showed that the average number of sexual behaviors with each sexual partner influenced the cost associated with 1 QALY saved, which meant that interventions aimed at people with more high-risk sexual behaviors gained more health economic benefits. This suggested that the intervention strategy of this study is suitable for high-risk MSM in China, which could lead to accurate allocation of prevention resources based on risk assessment. Meanwhile, if the one-size-fits-all intervention model does not match the risk level of the target population, it will easily lead to a waste of resources. Another important reason why this intervention strategy is cost-effective might be because the intervention was internet based, which could save on housing and office facility costs, among others. Therefore, the risk assessment–based integrated online intervention is not only low cost but also likely to reach MSM who may not have the time, resources, or motivation to seek prevention services, which would improve the convenience and accessibility of prevention resources.

### Limitations

There are several limitations in this study. First, the number of infections averted was estimated based on a model, and the model parameters were average values from the reported studies, which may have ignored the heterogeneity of data—the probability of HIV infection varies with the types of sexual partners—and may have impacted the estimated number of HIV infections averted. Further studies should distinguish the probability of HIV infection resulting from different subgroups, sexual roles, and sexual behavior patterns of MSM in order to accurately estimate the number of infections averted. Second, the number of sexual behaviors with each sexual partner was obtained from the literature and was not verified by questionnaires. Therefore, specific questionnaires need to be designed to investigate the number of sexual behaviors in order to test the reliability of input parameters.

### Conclusions

Our analysis demonstrates that a mobile phone–based digital intervention to prevent HIV infection among MSM is very cost-effective. These findings can inform public health officials’ and policy makers’ decisions about the selection of, and recommendations for, intervention measures. These findings can also help in the exploration of innovative HIV intervention strategies in order to more effectively allocate resources to the prevention of HIV among MSM.

## Abbreviations

CER: cost-effectiveness ratio
CUR: cost-utility ratio
GDP: gross domestic product
ICER: incremental cost-effectiveness ratio
MSM: men who have sex with men
QALY: quality-adjusted life-year
STI: sexually transmitted infection
